# Changes in the temporal distribution of in-hospital mortality in severely injured patients—An analysis of the TraumaRegister DGU

**DOI:** 10.1371/journal.pone.0212095

**Published:** 2019-02-22

**Authors:** Rauend Rauf, Francesca von Matthey, Moritz Croenlein, Michael Zyskowski, Martijn van Griensven, Peter Biberthaler, Rolf Lefering, Stefan Huber-Wagner, the Section NIS of DGU

**Affiliations:** 1 Department of Trauma Surgery, Technical University Munich, Hospital Rechts der Isar, Munich, Germany; 2 Institute for Research in Operative Medicine (IFOM), University of Witten/Herdecke, Cologne, Germany; University of Notre Dame Australia, AUSTRALIA

## Abstract

**Background:**

The temporal distribution of trauma mortality has been classically described as a trimodal pattern with an immediate, early and late peak. In modern health care systems this time distribution has changed.

**Methods:**

Data from the TraumaRegister DGU was analysed retrospectively. Between 2002 and 2015, all registered in-hospital deaths with an Injury Severity Score (ISS) ≥ 16 were evaluated considering time of death, trauma mechanism, injured body area, age distribution, rates of sepsis and multiple organ failure. Pre-hospital and post-discharge trauma deaths were not considered.

**Results:**

78 310 severely injured patients were registered, non-survivors constituted 14 816, representing an in-hospital mortality rate of 18.9%. Mean ISS of non-survivors was 36.0±16.0, 66.7% were male, mean age was 59.5±23.5. Within the first hour after admission to hospital, 10.8% of deaths occurred, after 6 hours the percentage increased to 25.5%, after 12 hours 40.0%, after 24 hours 53.2% and within the first 48 hours 61.9%. Mortality showed a constant temporal decrease. Severe head injury (defined by Abbreviated Injury Scale, AIS-Head≥3) was found in 76.4% of non-survivors. Patients with an isolated head injury showed a more distinct decrease in survival rate, which was accentuated in the first days after admission. The correlation of age and time of death showed a proportional increase with age (55-74a). The rate of sepsis and multiple organ failure among non-survivors was 11.5% and 70.1%, respectively.

**Conclusion:**

In a modern trauma care system, the mortality distribution of severely injured patients has changed its pattern, where especially the third peak is no longer detectable.

## Introduction

In their seminal studies published between 1974 and 1983, Trunkey and Baker proposed a trimodal distribution of trauma mortality based on a retrospective analysis of autopsy records of 425 and 437 fatalities, respectively [1–3]. The introduced pattern of temporal distribution distinguishes between three peaks: immediate deaths within minutes—usually at the scene due to nonsurvivable injuries—early deaths within hours of hospital arrival in the emergency department or the operating room due to severe but potentially survivable injuries and late deaths within weeks due to sepsis and related multiple organ failure.

In 1995, Sauaia et al. reassessed the epidemiology of trauma mortality based upon data of 289 patients and did not observe a trimodal temporal distribution, but a continuous decline of deaths which they contributed to improvements in trauma care systems [4]. In fact, a number of cofactors influencing the outcome of trauma patients have been substantially refined by advancements in injury prevention measures, prehospital care, implementation of standardized concepts like ATLS (Advanced Trauma Life Support), surgical techniques and strategies as well as intensive care therapies that led to a constant reduction in mortality rate [5, 6].

Since these hallmark publications, a series of studies addressing civilian trauma care and mortality distribution have been conducted [7–17]. However, most of them with a relatively small number of patients within a limited geographical dimension and with a wide range of time intervals making it difficult to determine a clear distribution.

This paper evaluates severely injured non-survivors who reached the hospital alive but then died, in terms of temporal distribution and injury patterns based on data of the TraumaRegister DGU. To our knowledge, it is the largest collective of severely injured patients analysed to date.

## Methods

In the present study, data is retrospectively analysed from the TraumaRegister DGU (TR-DGU) of the German Trauma Society (Deutsche Gesellschaft für Unfallchirurgie).

The TR-DGU was founded in 1993 and represents a multi-centre database with the aim of a pseudonymised and standardized documentation of severely injured patients. It not only serves as a quality assessment tool for the participating hospitals, but is also used for scientific evaluations of acute care. Datasets are registered from the time of admission and are structured prospectively in four consecutive phases from the site of the accident until discharge from hospital: A) Pre-hospital phase, B) Emergency room and initial surgery, C) Intensive care unit and D) Discharge. Documentation includes detailed information on demographics, injury pattern, comorbidities, pre- and in-hospital management, course on intensive care unit, relevant laboratory findings including data on transfusion and outcome of each individual. The inclusion criteria are admission to hospital via emergency room with subsequent ICU/IMC (intensive care unit/intermediate care unit) care or reaching the hospital with vital signs and death before admission to ICU. The infrastructure for documentation, data management, and data analysis is provided by the AUC—Academy for Trauma Surgery (AUC—Akademie der Unfallchirurgie GmbH), a company affiliated to the German Trauma Society. The scientific leadership is provided by the Committee on Emergency Medicine, Intensive Care and Trauma Management (Section NIS) of the German Trauma Society. The participating hospitals submit their data pseudonymised into a central database via a web-based application. Scientific data analysis is approved according to a peer review procedure established by Sektion NIS. 90% of the participating hospitals are primarily located in Germany, but a rising number of hospitals of other countries contribute data as well, currently from Austria, Belgium, China, Finland, Luxembourg, Slovenia, Switzerland, The Netherlands, and the United Arab Emirates). In the current study, only data from Germany are analysed. Since its foundation almost 25 years ago, datasets of almost 240,000 patients are collected in the TR-DGU. Currently, approx. 25,000 cases from more than 600 hospitals are annually entered into the database.

In Germany, the emergency medical system is carried out by specially qualified physicians who treat patients at the scene and perform interventions if necessary. Within the TraumaNetwork of the German Society of Trauma Surgery (*TraumaNetzwerk DGU*, TNW-DGU) the German trauma system is organised by regionalisation where hospitals are classified and certified as local, regional and supraregional trauma centres by specific requirements regarding personnel, infrastructure, admission capacity and responsibility [18]. In the regional trauma network it is the goal to have a maximum transportation time of 30 minutes from scene to trauma centre. The severely injured patient is primarily transported to a regional or supraregional trauma centre, if possible. If the anticipated transportation time exceeds 30 minutes, the patient will be admitted to a local trauma centre. Treatment procedures and transfer criteria in the early phase of care are based on the S-3 guidelines of the German Society of Trauma Surgery [19]. For hospitals associated with TNW-DGU the entry of at least a basic data set is obligatory for reasons of quality assurance. For non-registered hospitals participation in TraumaRegister DGU is voluntary. However, regarding the data collected between 2002 and 2015, the nationwide covering of hospitals by TNW-DGU constantly increased from 60 trauma centres in 2002 to 636 in 2015. Thus, data of the late investigation period is nearly representative for the whole trauma system in Germany [20].

Based on the methods published by our group [21], the data of severely injured patients who died in hospital were analysed in the period from 2002 to 2015. All injuries are coded using the Abbreviated Injury Scale (AIS) 2005 in a reduced version with 450 codes. Codes from the previous versions (AIS-98) were matched appropriately.

Patients who died in the prehospital setting (at the scene, during transport) as well as those who suffered from burns, drowning, poisoning, or hanging and also deaths after discharge are not documented in the TR-DGU. There is no follow-up of cases after discharge from acute care hospitals. Thus, the present analysis only focuses on in-hospital deaths. For inclusion criteria, please see Table 1.

**Table 1:** Inclusion criteria.

**Time period 2002–2015**
ISS ≥ 16
Died in acute care hospital
Date of admission and date of death available Only primary admitted cases; no transfer in / no early (2 days) transfer out Only Germany

Severely injured patients were characterized by an ISS ≥ 16 [22].

For inclusion, the date of admission to the acute care hospital and the date of death were required.

Since the temporal distribution of trauma mortality may basically be a function of the selected time interval model [12], we merely subdivided the time intervals of death into hours, days and weeks.

The analysis of the time to death had to be performed only from the time of primary admission (under exclusion of transfer in/early out) in order to obtain a homogeneous data set.

The time data correspond to the time after admission according to the TR-DGU calculation program: The hour of death was 0 if the patient died within the first 60 minutes after admission. The hour of death was 1 if the patient died between 60 and 120 minutes. The day of death was 1 if death occurred between 24 and 48 h after admission. If the time of admission and/or death was unknown, the time of death was defined as the difference between the date of admission and the date of death.

Besides mortality and its temporal distribution, further analyses included demographic data, trauma mechanism, injured body region, transport time to hospital and length of stay at ICU. Furthermore, the rate of sepsis and multiple organ failure (MOF) among non-survivors were evaluated. The definition of sepsis and MOF is based on the SOFA-Score postulated by Vincent et al. [23] and is outlined in the TR-DGU standard documentation form.

The information was available in the standard documentation form only, used in 62% of all cases.

The precise cause of death is not yet implemented in the documentation of the TR-DGU.

The data were presented as means and percentages and are normally distributed. A Kaplan–Meier curve was used to describe the cumulative survival during the first 30 days in association with the injury subgroup. The time of death was also presented as the median for different conditions. As described by Lefering et al. [21], statistical testing was avoided, as the large number of cases would make even minor differences statistically significant.

### Ethics statement

The study received full approval from the ethics committee of the medical faculty of Technical University of Munich, Germany (project number 62/17 S). The present study is in line with the publication guidelines of the TraumaRegister DGU (TR-DGU project ID 2015–031).

The authors confirm that no consent from patients to participate was needed because of the purely retrospective study design. Patient data were pseudonymised for analytic purposes.

## Results

In the period from 2002–2015, 78 310 severely injured patients with an ISS ≥ 16 were documented in the TR-DGU. 14 816 of these patients died in hospital, which constitutes a hospital mortality rate of 18.9%.

Mean ISS of non-survivors was 36.0 ± 16.0, compared to 25.2 ± 9.3 in survivors.

**Fig 1:**
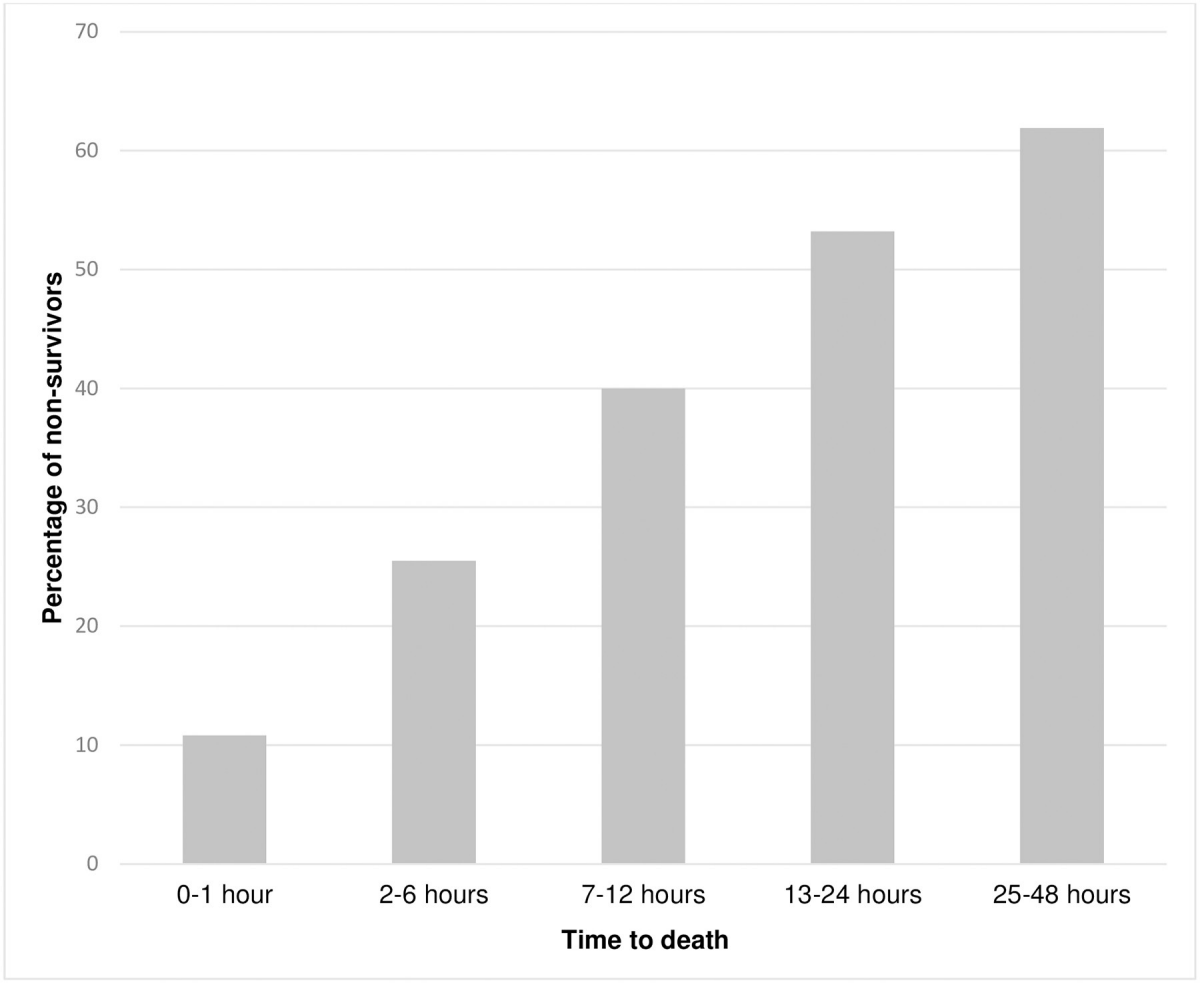
Percentage of in-hospital fatalities within the first 48 hours after admission.

**Fig 2:**
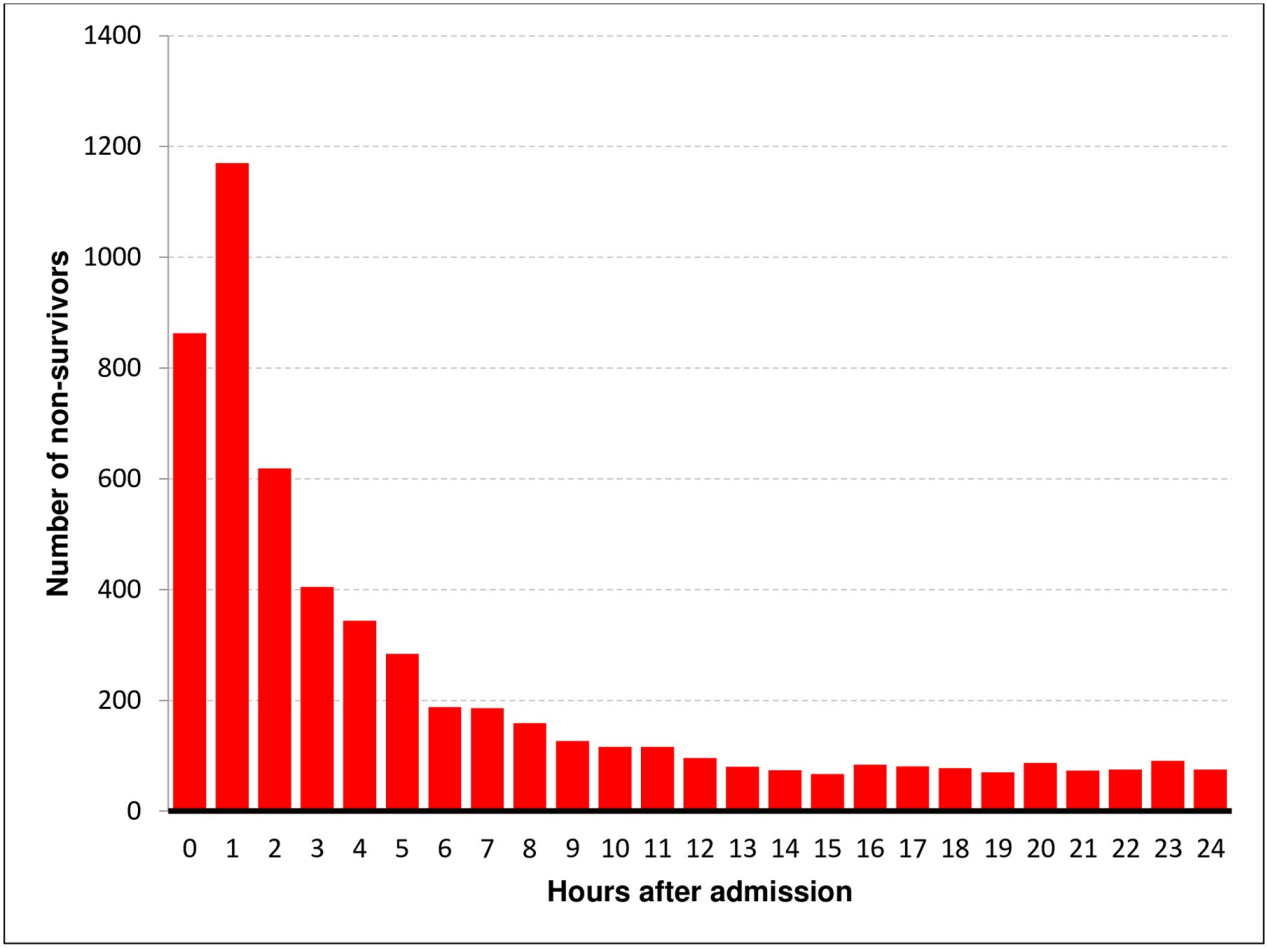
Temporal distribution of in-hospital deaths in hour intervals.

Within the first hour after admission, 10.8% of deaths occurred, after 6 hours the percentage increased to 25.5%, after 12 hours 40.0% of the non-survivors had died, after 24 hours more than half of all fatalities had already occurred (53.2%), and nearly two thirds of all deaths occurred within the first 48 hours (61.9%), as presented in Fig 1. For the time distribution of death in hour intervals, please see Fig 2.

**Fig 3:**
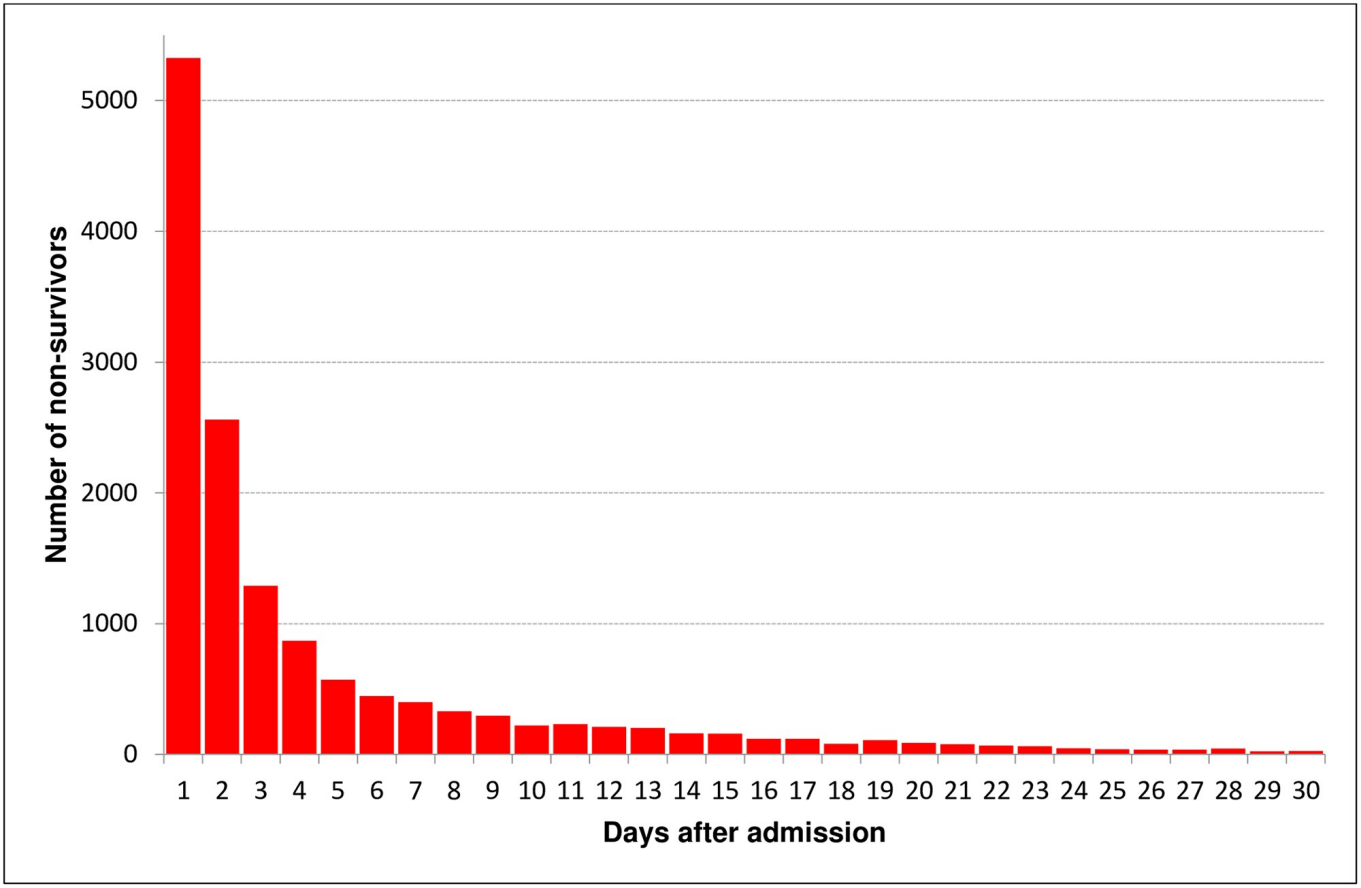
Temporal distribution of in-hospital deaths in day intervals.

**Fig 4:**
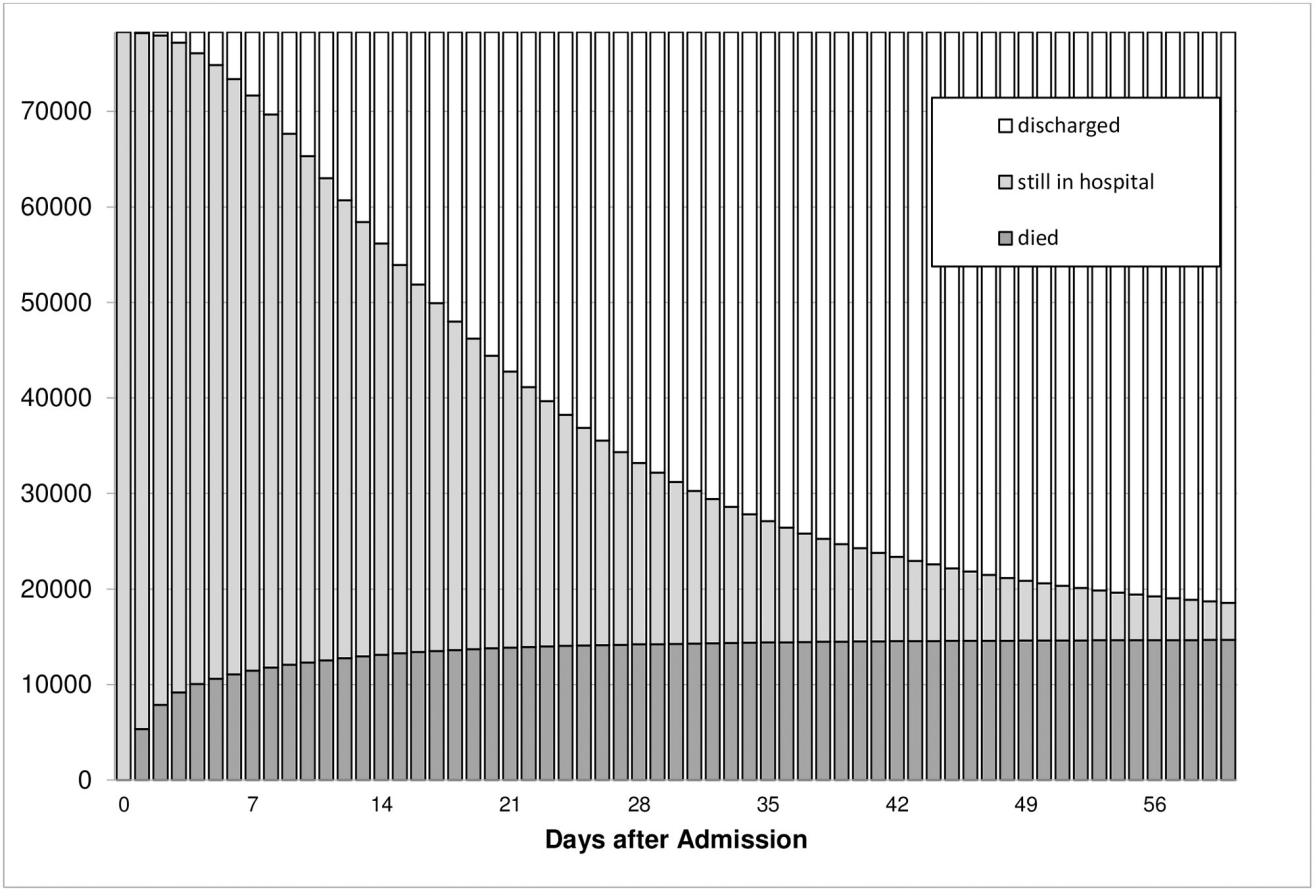
Cumulative number of severely injured patients after admission to acute care hospital.

Fig 3 shows the temporal distribution of deaths in day intervals, also depicting a lack of a bi- or trimodal pattern. Similarly, Fig 4 illustrates an initial linear increase in the number of non-survivals which reaches a stable state within the first few days after admission. Non-survivors had a mean time to death of 6.5 days (median 2 days).

**Fig 5:**
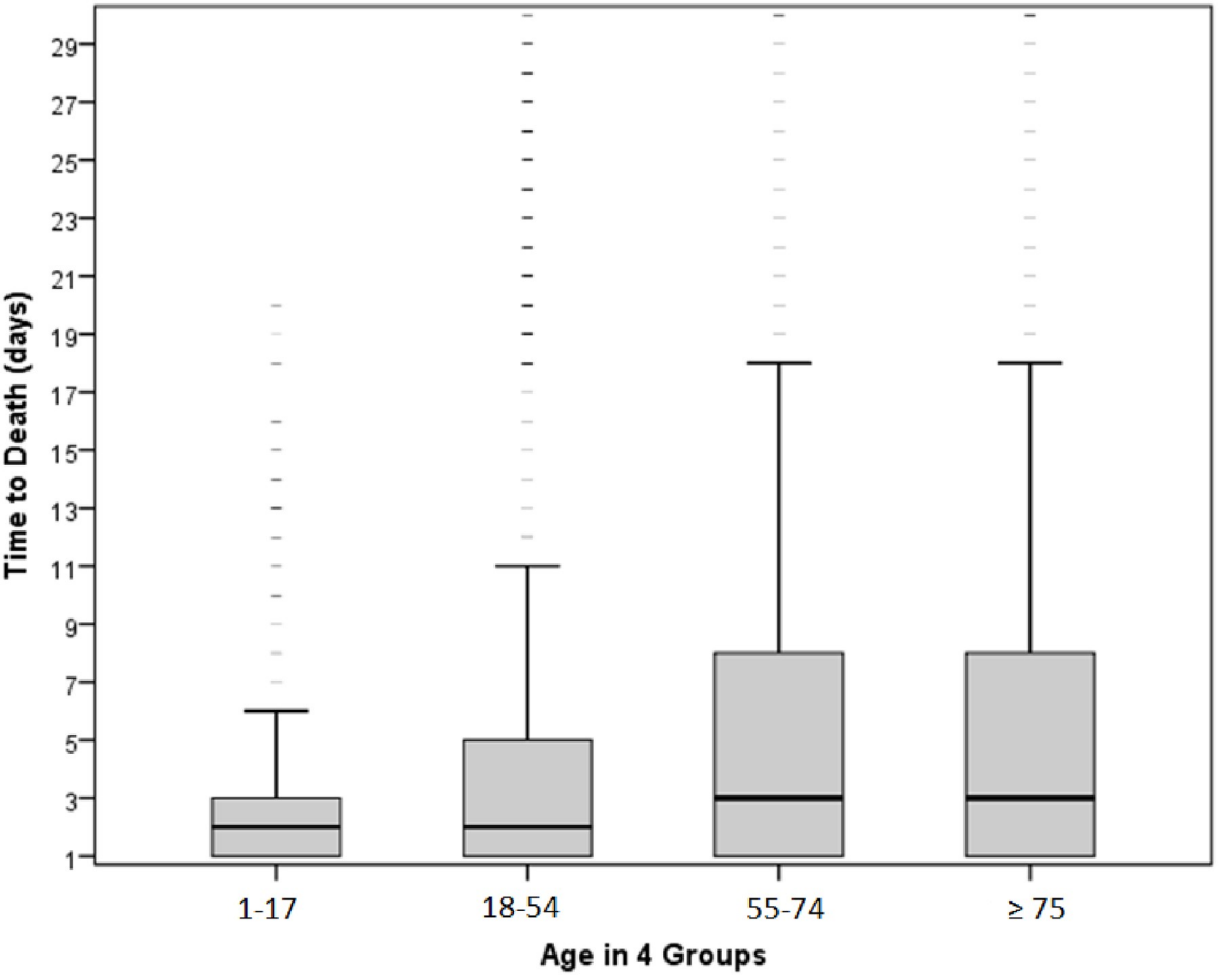
Correlation between age and time of death. Non-survivors were divided into 4 subgroups.

67% of the non-survivor group were male and had a mean age of 59.5 ± 23.5 years (survivors 47.3 ± 20.9 years). 2.4% of the non-survivors were under 16 years old. The correlation of age and time of death showed a proportional increase with age. Non-survivors were grouped in 4 subgroups based on their age: 1–17, 18–54, 55–74 and ≥ 75 years, respectively. The youngest group had a mean time to death of 3.9 days, the 18–54 group 5.2, the 55–74 group 8.0 and the ≥ 75 group 6.9 days (Fig 5); the respective medians were 2, 2, 3, and 3 days.

**Fig 6:**
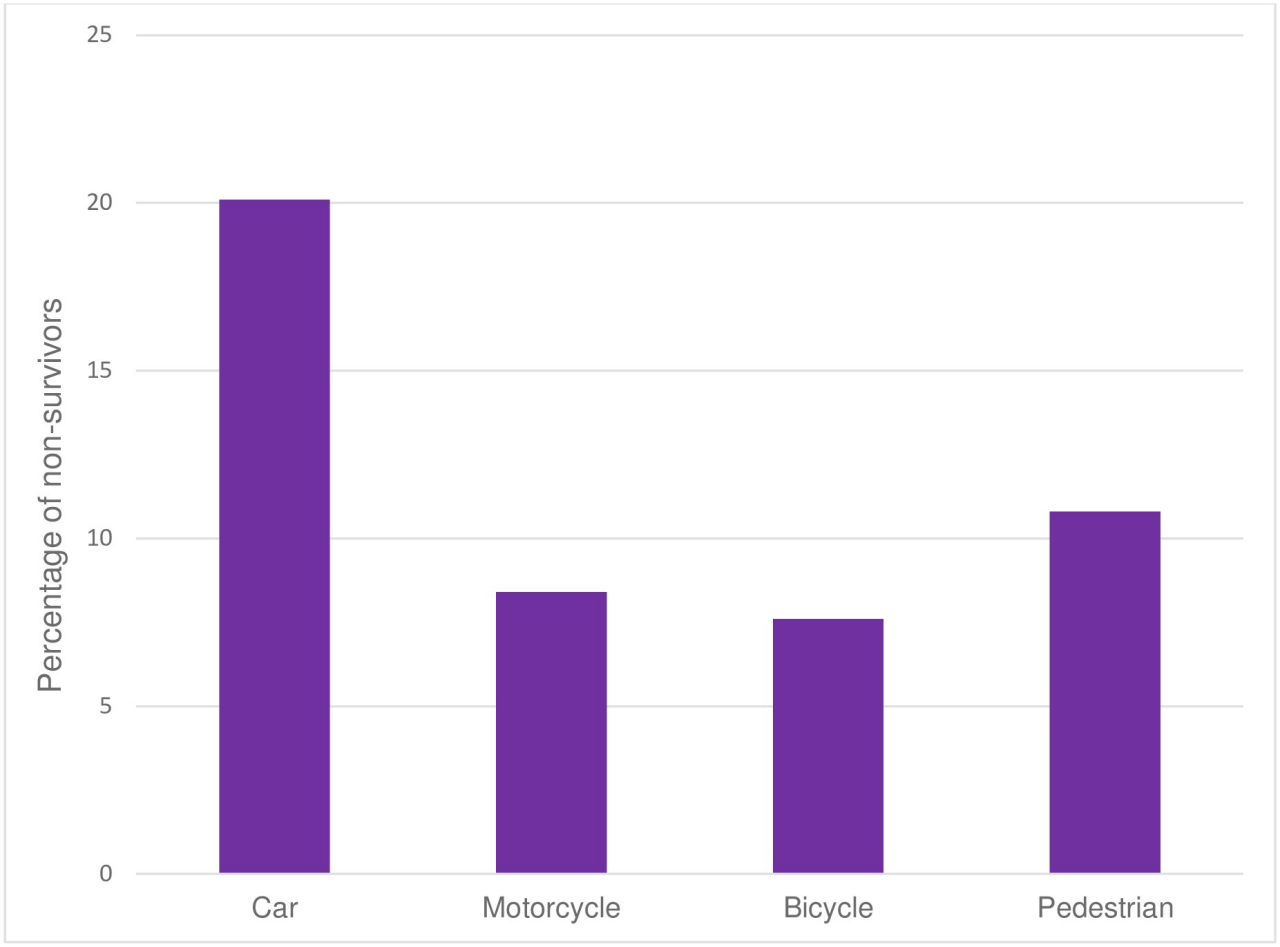
Percentage of traffic accident modalities as leading cause of fatal trauma.

The main trauma mechanism in the analysed cases was blunt trauma in about 95%. Traffic accidents accounted for 47.0% of deaths with car accidents as leading cause (Fig 6). 18.1% died of a high falls (> 3m) and 26.0% suffered from fatal low falls (< 3m).

**Fig 7:**
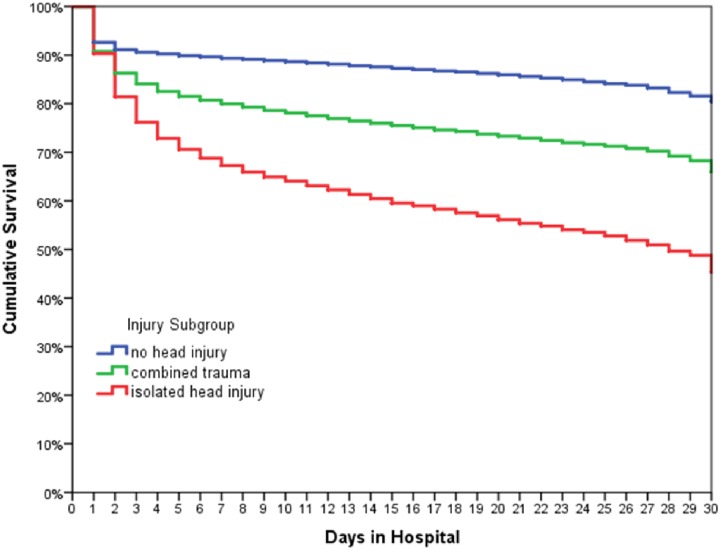
Kaplan–Meier curve for cumulative survival, correlated to three injury subgroups. Number of patients involved: n = 78 310 severely injured patients with ISS ≥ 16; 14 816 non-survivors.

Severe head injuries, as defined by an AIS-Head ≥ 3, were found in 76.4% of non-survivors and 47.2% of survivors. 69.2% of the non-survivors had an initial Glasgow Coma Scale of ≤ 8 versus 20.1% of the survivors. The Kaplan-Meier curves for the first 30 days in the injury subgroups (1) no head injury, (2) isolated head injury and (3) combined trauma, showed that patients with isolated head injuries had a more distinct decrease in survival rate. This was mainly based on differences in the first days after admission (Fig 7). Non-survivors with isolated head injuries were on average 10 years older than the deceased in the other two groups.

**Fig 8:**
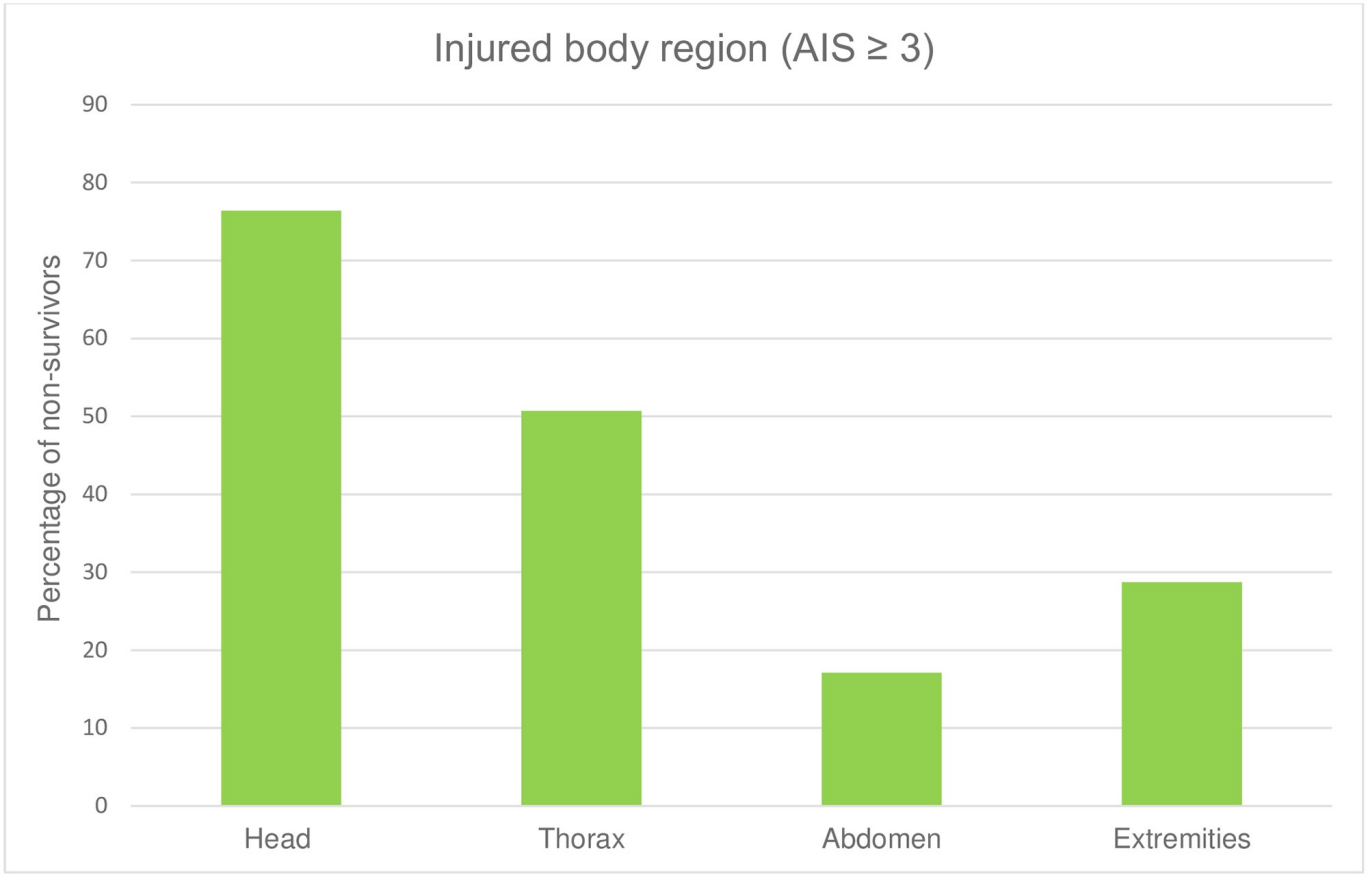
Percentage of trauma fatalities in correlation to injured body region.

50.7% of the fatalities had a thorax trauma with an AIS ≥ 3, 28.7% had an extremity trauma AIS ≥ 3 and 17.1% had a severe abdominal trauma AIS ≥ 3 (Fig 8).

A further parameter analysed was the period between accident and arrival at the acute hospital (total prehospital time) which averaged 66.3 ± 29.5 minutes for non-survivors and 65.3 ± 28.7 minutes for survivors.

Trauma fatalities were treated at the intensive care unit for a mean of 5 days, whereas survivors remained at ICU for 11 days on average. Non-survivors remained intubated for a mean of 4.3 days (median 1 day), severely injured survivors had an intubation time of 5.8 days (median 1 day).

The rate of sepsis and multiple organ failure among non-survivors was 11.5% and 70.1%, respectively. The mean/median time to death in cases with sepsis was 23.3 / 17 days. Non-survivors with MOF showed a mean/median time to death of 8.7 / 4 days. If only cases with time to death > 3 days were considered, then the sepsis rate would be 23.1% (n = 747 of 3 234).

## Discussion

The analysis of trauma mortality and its temporal distribution is crucial for the development and improvement of trauma care systems. In our present study, we could demonstrate that the time distribution of death in severely injured patients does not follow a tri- or bimodal pattern.

The studies by Trunkey and Baker conducted in the 1970’s in San Francisco were based on data from autopsy records of 437 and 425 trauma deaths, respectively [1, 3]. In a subsequent review, they proposed the often cited “trimodal distribution” of trauma fatalities, with more than half of the deaths occurring in the pre-hospital setting, which represents the first peak (“immediate”, within 60 minutes), usually due to not-survivable injuries of the central nervous system (CNS) and major vascular trauma [24]. More recent papers also describe a still high pre-hospital mortality rate [8, 25–27]. However, the data vary from 30 to 70%, which is attributed to various factors such as injury mechanism, body area, age of patient and also geographic and logistic issues. As the present study is based on in-hospital data only, this aspect of trauma mortality could not be addressed. However, linking the present data with pre-hospital mortality distributions as mentioned above depicts a bimodal pattern, considering that in the current study a third peak was not detectable.

The second so-called peak represented the early hospital deaths that occurred a few hours after injury and constituted about 34% of fatalities, mostly due to brain injury and exsanguination. The third peak (about 20%) consisted of the late deaths (> 1 week), mostly due to sepsis and multiple organ failure.

Several papers published subsequently found the absence of a trimodal distribution, but rather bimodal or unimodal patterns. [4, 8, 27–29]. Accordingly, our present analysis shows a continuous decrease in mortality. From those cases with a fatal outcome who reached a hospital, over half of them had already died 24 hours later, and after 48 hours nearly two thirds, which is consistent with previously published data [16, 26].

Several authors also questioned the trimodal pattern and suggested that the temporal distribution is an effect of the selected time interval model [12, 30]. We therefore omitted a subdivision of time intervals into strict phases such as “early” (hours), “intermediate” (days) or “late” (weeks). Independent of such groups, we found the mortality rate to constantly decline with time.

The present study focusses on severely injured patients with a mean ISS of 36. In a recent investigation Valdez et al. analysed a large collective of trauma patients based on data of a national trauma registry in the US [31]. Between 2002 and 2006 the authors report on nearly 900 000 trauma patients, however, with a mean ISS of 11 ± 10, therefore including a large number of light injuries. Nonetheless, the time distribution pattern of death was comparable to the current data.

In a previous paper, Lefering and co-workers published combined data of two large registries (TR-DGU and Trauma Audit and Research Network (TARN), UK) [21]. Based on a total of 43 958 cases, they identified 6 685 and 6 867 in-hospital fatalities, respectively, and also found the absence of a bimodal mortality distribution. However, the data was not homogenous (two different registries, 932 patients in TARN with missing time of death) and the injury mechanisms and patterns were not directly analysed. In the present study, we analysed injury mechanisms and body regions involved and found blunt trauma to be the most frequent force in a Western European country (about 95%), which corresponds to other European studies [12, 16]. Thus, motor vehicle accidents and falls still are the main cause of trauma fatalities in Germany. In a large study of trauma deaths in Los Angeles which also included pre-hospital fatalities, Demetriades et al. not only showed that there is “no specific” temporal distribution of mortality, but also postulated that the distribution of deaths highly depends on mechanisms of injury, the injury patterns, severity and age of the patients [26]. This analysis also showed that the temporal distribution of death in penetrating trauma is different from that in blunt trauma. However, both mechanisms, blunt and penetrating trauma, presented with two distinct peaks, pre-hospital and early in-hospital (1–6 hours). After the latter peak, a constant and continuous decrease in mortality with no signs of a trimodal distribution was observed. This continuous decline pattern is consistent with our results when focussing on in-hospital deaths.

In our study, severe head injury (AIS ≥ 3, GCS ≤ 8) was found in nearly 70% of non-survivors, indicating the head to be the most frequently injured body region associated with traumatic death, especially in the early setting after admission. This is comparable to other studies [4, 16, 26]. However, patients with an isolated head injury showed a more distinct decrease in survival rate than those without or with combined injuries. A possible explanation for this finding is that we included patients with an ISS ≥ 16 implying that patients with an isolated head injury had a more severe TBI than those with combined lesions. Furthermore, non-survivors with an isolated head injury were on average 10 years older than the deceased in the other two groups.

50% of the non-survivors sustained a severe thorax trauma followed by extremity injuries (nearly 30%) and abdomen trauma (17%). This is comparable to European studies [12], but not fully to U.S. data, where gunshot wounds are more frequent: there, in contrast to our results, abdominal trauma shows higher rates than extremity injuries [4, 26].

An overall reduction in trauma mortality in the last decades has been observed, presently with an average of 18% in polytrauma patients [5, 6, 32], which is confirmed by our current data (18.9%). Since the description of trimodality over 40 years ago, several advancements in trauma prevention, rescue and treatment concepts, such as advanced trauma life support (ATLS) or damage control strategies, diagnostic algorithms, such as the implementation of focused assessment with sonography for trauma (FAST) and CT scans in the ER, as well as further developments in intensive care medicine, have contributed to a reduction in trauma mortality [32]. Whether these improvements have led to a direct modification of the temporal distribution could not be proved to date [33]. Furthermore, the differences between present trauma systems and the time point of analysis, as well as geographic and demographic factors also might influence the temporal distribution of fatal trauma [4, 7, 8]. For example, an Indian analysis by Sahdev et al. reported an additional fourth peak between day one and two, which was titled “delayed deaths” [10]. This demonstrates that trauma mortality patterns are also a function of the trauma systems they are analysed in.

Soreide et al. argued whether the time of death in trauma victims may merely be an effect of selected time interval models and proposed that it should be used as an educational tool [12].

In the present study, the correlation of age and time of death showed a proportional increase with age which is comparable to other demographic data [12]. Above the age of 75 the time to death decreased again. We did not evaluate ISS in relation to specific age groups, for it was not the purpose of this study. However, age is a prognostic factor for a higher trauma-associated mortality independent of ISS, as reported by Kuhne et al. [34]. It seems to be an adequate explanation that older non-survivors have a lower trauma load than younger ones, which nonetheless is fatal due to age-related comorbidities and a reduced capacity to recover, as described by other authors [12, 16, 35]. In a prior analysis of register datasets, Lefering et al. could show that patients who die beyond day 30 as a consequence of their injuries are much older than patients who died earlier [21].

Sepsis and MOF still are severe complications after trauma [36, 37]. In the current study, the rate of sepsis and MOF among non-survivors was 11.5% and 70.1%, respectively. In a retrospective study on MOF in multiple trauma patients, our group observed a constant decrease in mortality, however, this was accompanied by a significant increase in the incidence of MOF [38]. Sepsis and MOF are the leading cause of late death (> 1 week), as described by several authors [1, 4, 12]. This is consistent with the results of the current investigation, where the mean/median time to death in cases with sepsis was 23.3 / 17 days. If only cases with time to death > 3 days were considered, the sepsis rate would be 23.1%.

In the present investigation, the total prehospital time did not differ between survivors and non-survivors and averaged 65 minutes. In a recently published review by Harmsen et al., a distinct correlation between total prehospital time and reduction in mortality could not be made–times slightly higher than sixty minutes tend to be associated with a better outcome, however, the data collected is heterogeneous [39].

Limitations: A limitation of the current investigation is that the registry data analysed only includes in-hospital trauma fatalities, excluding victims that died at scene or during transport and therefore could not be included in the TR-DGU. Furthermore, there is no follow-up of cases after discharge from acute care hospitals, therefore the study lacks data on post-discharge mortality. This may lead to an underestimation of the total number of deaths in the first hour after trauma. However, as described by several authors who also included deaths in the pre-hospital setting, more than half of all fatalities occur before arrival at the emergency department [12, 17, 25]. This is not contrary to but rather in support of the pattern of a continuously decreasing mortality rate when compared to our in-hospital data, where more than half of all deaths occur within the first 24 hours. Linking our present data with the pre-hospital mortality distributions analysed by other authors depicts a bimodal pattern, considering that in the current study a third peak was not detectable.

## Conclusion

The analysis of a large dataset from the TraumaRegister DGU on in-hospital trauma fatalities shows a continuous decrease in incidence after admission with more than half of all deaths occurring within the first 24 hours. Patients with an isolated head injury showed a more distinct decrease in survival-rate in the first days after admission. Higher age (55–74) is associated with a longer time to death, however, above 75 years of age the time to death decreases again.
